# Successful treatment of advanced lung adenocarcinoma complicated with Lambert‐Eaton myasthenic syndrome: A case report and literature review

**DOI:** 10.1111/1759-7714.13385

**Published:** 2020-03-10

**Authors:** Aili Wang, Xin Zhang, Jiawen Yi, Min Zhu, Yuhui Zhang

**Affiliations:** ^1^ Department of Respiratory and Critical Care Medicine, Beijing Luhe Hospital Capital Medical University Beijing China; ^2^ Department of Tuberculosis Shijiazhuang Fifth Hospital Hebei China; ^3^ Department of Respiratory and Critical Care Medicine, Beijing Chao‐Yang Hospital Capital Medical University, Beijing Institute of Respiratory Medicine Beijing China

**Keywords:** Adenocarcinoma of lung, Lambert‐Eaton, myasthenic syndrome

## Abstract

Lambert‐Eaton myasthenic syndrome (LEMS) is a rare disease characterized by involvement of the neuromuscular junction. Most cases have an underlying malignancy, especially small‐cell lung cancer (SCLC), while adenocarcinoma is less common. Here, we report a rare case of metastatic lung adenocarcinoma complicated with LEMS. In this case, L858R mutation was detected in the 21st exon of the *EGFR* gene. First‐line treatment with gefitinib was given, and the patient has survived for more than six years. Early diagnosis of LEMS and timely and effective treatment can result in a good prognosis. We also searched for “lung cancer”, or “carcinoma of lung”, or “adenocarcinoma of lung”, or “Lambert‐Eaton myasthenic syndrome” in PubMed until 1 December 2019. Seven cases of lung adenocarcinoma complicated with LEMS were found, most of which had a poor prognosis.

**Key points:**

This article reports a rare case of metastatic lung adenocarcinoma with *EGFR* mutation, complicated with LEMS. Gefitinib was given as first‐line treatment, and resulted in a good prognosis.

## Introduction

Lambert‐Eaton myasthenic syndrome (LEMS) is an autoimmune neuromuscular junction disorder. Antibodies against presynaptic voltage‐gated calcium channels are produced in patients with LEMS, decreasing the calcium that can enter the nerve ending, hence the neuromuscular junction releases less acetylcholine.^1^ Around 50% to 60% of patients with LEMS have an underlying malignancy, regarded as a special type of paraneoplastic syndrome, known as tumor associated LEMS (T‐LEMS). Small‐cell lung cancer (SCLC) is the most common in T‐LEMS. Patients without cancer after a lengthy period are regarded as nontumor‐related LEMS (NT‐LEMS). The mechanism which causes the cancers and lack of cancers is not clear.^2^ Few cases of lung adenocarcinoma complicated with LEMS have been reported. This article describes such a case who has remained in a stable condition and survived for more than six years.

## Case report

**Figure 1:**
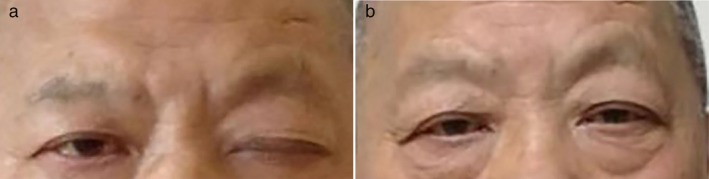
(**a**) Drooping of the left upper eyelid (December 2013). (**b**) The left upper eyelid was back to normal (6 January 2014).

A 63‐year‐old smoking male was admitted to Beijing Chao‐Yang Hospital, Capital Medical University, on 12 November 2013 with a history of his left eyelid drooping without obvious cause for 10 days (Fig 1). The symptoms were more serious in the evening than morning, and complicated by blurred vision and diplopia. Past medical history included hypertension, diabetes mellitus, left facial neuritis, and pacemaker implantation.

**Figure 2:**
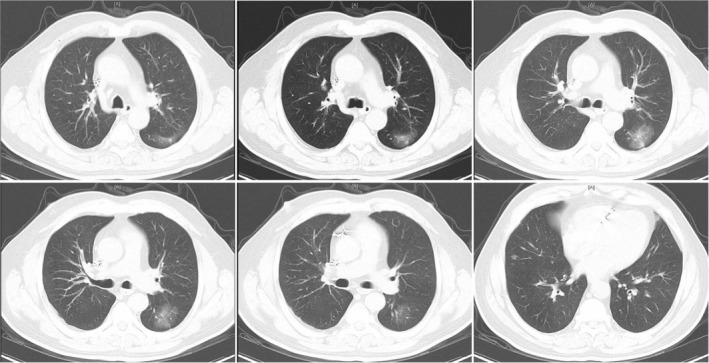
Blur nodules in the dorsal segment of the lower lobe of the left lung (19 Nov 2013).

Physical examination showed a symmetrical frontal stripe, he was unable to frown and his left eyelid drooped. Blood test results showed normal erythrocyte sedimentation rate, tuberculin test, C reactive protein, anti‐dsDNA antibody, anti‐guaninine peptide antibody, and antineutrophil cytoplasmic antibodies. Tuberculosis IgG/IgM antibody detected by colloidal gold method was positive, and antinuclear antibodies revealed S1:100. A chest CT scan was subsequently performed which revealed a left lower lobe nodule with nonuniform density and pleural retraction (Fig 2). A needle biopsy verified that it was adenocarcinoma. Gene detection of *EGFR* showed L858R mutation. Bone scan revealed multiple bone involvements.

Test for paraneoplastic syndrome antibodies of anti‐Hu, anti‐Ri, anti‐Yo were negative. Neostigmine test, anti‐acetylcholinesterase antibody, and muscle‐specific tyrosine kinase antibody were also negative. Electromyography examination was not conducted because of the patient's pacemaker.

The final diagnosis concluded that the patient had stage IV (T4, N3, M1b) left lower lobe lung adenocarcinoma, with left hilar lymph node, right mediastinal lymph node, right lung and multiple bone metastasis. The left eyelid drooping was an indication of LEMS as a result of adenocarcinoma.

**Figure 3:**
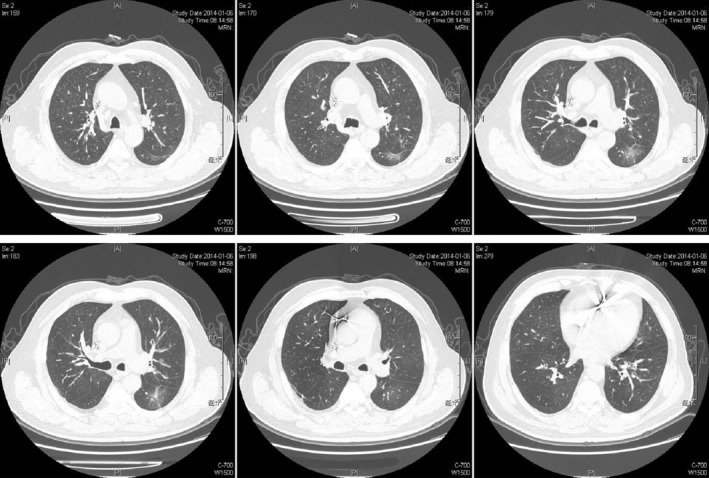
A chest scan was taken one month after gefitinib treatment, which indicated a reduction in size of the blur nodules.

The patient was subsequently treated with gefitinib which commenced on 7 December 2013. One month following his treatment, his left eyelid drooping symptom began to resolve (Fig 1). Chest scan on 6 January 2014 displayed a reduction in size of the left lung nodules (Fig 3). Follow‐up CT examination was conducted every two to three months. Bone scan, abdominal ultrasound, and head MRI were examined every six months. The patient has remained in a stable condition with neither recurrence of metastasis nor left eyelid droop for more than six years.

We found seven similar cases of lung adenocarcinoma with LEMS (Table 1)^3^, ^4^, ^5^, ^6^, ^7^, ^8^, ^9^. All cases were diagnosed at an advanced stage, and only two of them were alive at the time of publication of the articles.

**Table 1:** Cases with lung adenocarcinoma complicated with LEMS in 1987—2019

References	Year	Cases	Gender	Age	Clinical manifestations	Imaging manifestations	Biopsy	Treatment	Outcome
Bukhari *et al* 3	2017	1	Female	78	Leg weakness	Mass in right upper lung	Percutaneous biopsy	Palliative radiation therapy	Death
Arai *et al*.^4^	2012	1	Male	75	Leg weakness	Mass in right lower lung	Right lower lobectomy	Operation and palliative care	Unknown
Milanez *et al*.^5^	2008	1	Male	66	Dysphagia and extensor weakness	Shadow of the apex nodules of the right lung	Right upper lobectomy	Operation and palliative care	Death
Chang *et al*.^6^	2013	1	Male	73	Ptosis, dysphagia and extensor weakness	Pretracheal regional lymph node enlargement	Thoracoscope	Unknown	Unknown
Tang *et al*.^7^	2017	1	Male	65	Ptosis and diplopia	Mass in left lower lung	Left lower lobectomy	Pyridostigmine bromide, steroid and operation	Alive
Ramos‐Yeo *et al*.^8^	1987	1	Male	56	Extensor weakness	Consolidation of the lower right lung	Autopsy	Palliative care	Death
Cai G *et al*.^9^	2019	1	Female	65	Dysarthria, ataxia and autonomic dysfunction	Bilateral pneumonic tumors	Bronchoscope‐guided biopsy	Chemotherapy and intravenous immunoglobulin	Alive

## Discussion

The main clinical manifestations of LEMS are symmetrical limb proximal muscle weakness, decreased or absent tendon reflex, and autonomic nervous dysfunctions. Muscle weakness often starts in the lower limbs, with more serious symptoms in the morning than the evening. Activities have been reported to reduce the symptoms.

The patient's first symptom was ophthalmoplegia, which is rare in LEMS, and no electromyogram was performed due to the patient having a pacemaker. It is hard to identify with myasthenia gravis. As a result, this case was discussed by specialists in multiple disciplines, including neurology, electromyography, oncology, respiratory and cardiology. It was agreed that myasthenia gravis is a recurrent and progressively worsening disease, but after antitumor treatment, the patient has no recurrent ophthalmoplegia for more than six years, so a diagnosis of T‐LEMS was considered. Only 5% of patients with LEMS are diagnosed with cancer, and about 86% of the patients have LEMS symptoms before their tumor appears,^10^ thus a diagnosis of NT‐LEMS should not be made easily, even if no tumor was found.

Few cases of lung adenocarcinoma complicated with LEMS have been previously reported. A PubMed literature search revealed seven published cases (Table 1), which were diagnosed at an advanced stage, with a poor prognosis. The patients ranged from 56–78 years of age (mean age, 68.3 ± 7.0 years). Six patients with adenocarcinoma diagnosed by biopsy, and one patient reported by Ramos‐Yeo and Reyes^8^ were diagnosed by autopsy seven years after they had been diagnosed with LEMS. A lung resection was performed in three out of seven cases, of which two died of postoperative complications. Only two patients were still alive when the articles were published.

Effective antitumor therapy is crucial for T‐LEMS. The *EGFR* gene mutation rates are 40% to 50% in Asian and Chinese patients with lung adenocarcinoma.^11^, ^12^ Multiple randomized stage III clinical studies^13^, ^14^, ^15^, ^16^, ^17^, ^18^, ^19^, ^20^ have revealed that treatment with EGFR‐TKIs (gefitinib, erlotinib, ectinib, afatinib, etc) improves progression‐free survival. In this case with a sensitive *EGFR* mutation, gefitinib was given as first‐line treatment, and the patient has survived for more than six years. Early diagnosis of LEMS and timely and effective treatment can bring a good prognosis.

LEMS is a rare disease, most diagnoses are associated with cancer, but it is extremely rare in lung adenocarcinoma. Relevant examinations are necessary for tumor screening in suspected LEMS patients. For T‐LEMS, treatment should be given to improve the prognosis, while for NT‐LEMS, a long period of follow‐up should be done to avoid occult malignancy.

## Disclosure

No authors report any conflict of interest.
